# A Major Complication in Micropercutaneous Nephrolithotomy: Upper Calyceal Perforation with Extrarenal Migration of Stone Fragments due to Increased Intrarenal Pelvic Pressure

**DOI:** 10.1155/2015/792780

**Published:** 2015-07-14

**Authors:** Fatih Akbulut, Burak Ucpinar, Metin Savun, Onur Kucuktopcu, Faruk Ozgor, Abdulmuttalip Simsek, Gokhan Gurbuz

**Affiliations:** Department of Urology, Haseki Training and Research Hospital, 34096 Istanbul, Turkey

## Abstract

Micropercutaneous nephrolithotomy is a safe and efficient technique for appropriate sized stones. It is performed through a 4.85 Fr all-seeing needle and stones are fragmented into dust, without the need for tract dilatation, unlike other percutaneous nephrolithotomy types. Even though micropercutaneous nephrolithotomy has many advantages, increase in intrapelvic pressure during surgery may cause rare but serious complications. Herein we report a case of micropercutaneous nephrolithotomy in a 20-year-old woman with a 20 mm right renal pelvis stone and present an undesired outcome of this complication, upper calyceal perforation. Right lower calyceal access was performed with 4.85 Fr all-seeing needle and 2 cm renal pelvis stone was fragmented by 272 *μ*m Holmium-Yag laser system. Upper calyceal perforation and infrahepatic accumulation of stone fragments were detected by fluoroscopy during the surgery. Postoperative imagings revealed perirenal urinoma, perirenal and infrahepatic stone fragments, and lower calyceal stone fragments inside the system. On second postoperative day, minipercutaneous nephrolithotomy and double J catheter insertion procedures were applied for effective drainage and stone clearance. Risk of calyceal perforation and urinoma formation, due to increased intrapelvic pressure during micropercutaneous nephrolithotomy, should be kept in mind.

## 1. Introduction 

Kidney stones can be treated in a variety of ways and percutaneous nephrolithotomy (PNL) has been a very important part of this treatment modalities since it was first introduced in 1976 [1].

After its first introduction, PNL method has undergone many improvements. Mini-PNL, ultramini-PNL, and micro-PNL are different types of PNL surgery which are commonly used in daily practice. These methods mainly vary in the sheath that is used during surgery. Since micro-PNL was first introduced in 2011, a lot of advantages and disadvantages of this method have been cited in several articles [2]. Micro-PNL is performed through a 4.85 Fr all-seeing needle, which is remarkably narrower than other sheaths that are used in other PNL types. This feature of micro-PNL causes less hemoglobin drop when compared with other PNL types [3]. Also, high probability of terminating the operation with a tubeless fashion, less hospital stay, less invasiveness, and no need of tract dilatation during surgery can be listed as advantages of this new technique [3]. There are also certain disadvantages of micro-PNL. High likelihood of blurred vision during surgery, increased intrarenal pelvic pressure (IPP) due to ineffective drainage of the system, risk of steinstrasse formation, and being an invasive procedure when compared to extracorporeal shock wave lithotripsy (ESWL) can be listed as disadvantages of micro-PNL. In this case report, we present a micro-PNL procedure that we performed, which ended up with a rare complication of micro-PNL, which is due to increased IPP during surgery.

## 2. Case Presentation 

**Figure 1:**
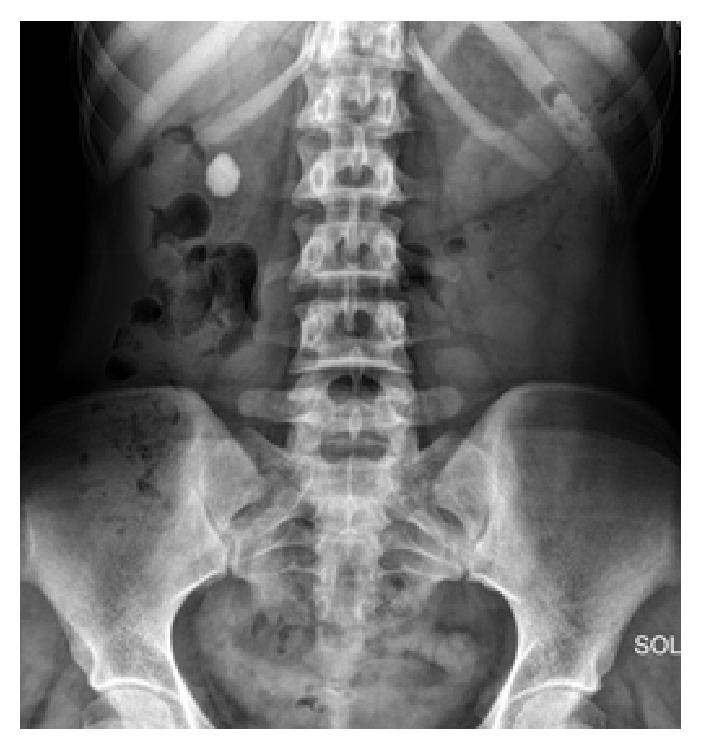
Preoperative kidney, ureter, and bladder X-ray of patient.

**Figure 2:**
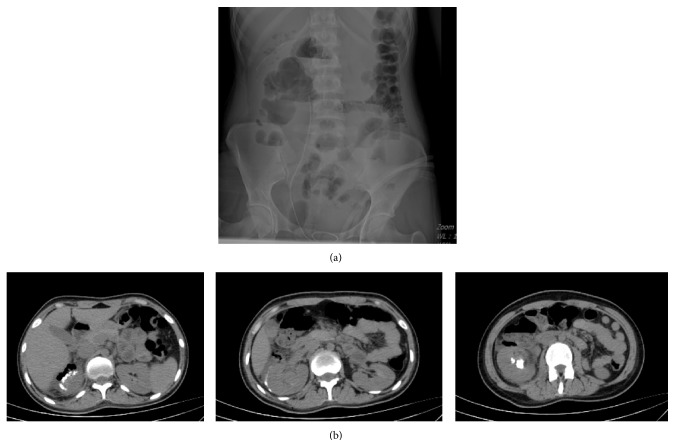
(a) Kidney, ureter, and bladder X-ray in postoperative day 1. (b) Perirenal urinoma, perirenal and infrahepatic stone fragments, and lower calyceal residual stone fragments were seen on computed tomography in postoperative day 1.

**Figure 3:**
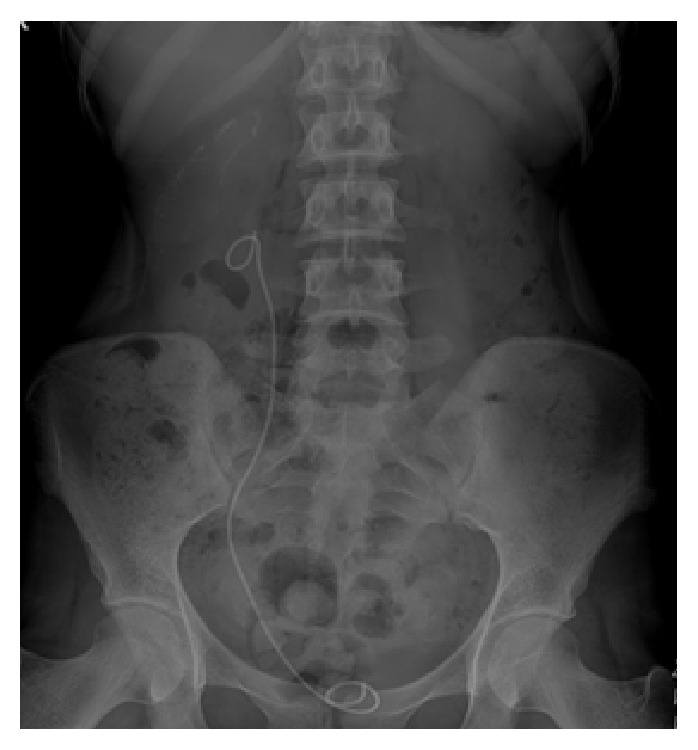
The right kidney was stone-free and perirenal collection of stone fragments was visible on kidney, ureter, and bladder X-ray after the second operation.

A 20-year-old woman referred to our clinic with right lumbar pain and there was no costovertebral angle tenderness on physical examination and all her vital signs were normal. 2 cm right renal pelvis stone was identified with noncontrast computed tomography and intravenous pyelography (Figure 1). Micro-PNL was planned for the patient. Under general anesthesia and in the lithotomy position, 5F ureteral catheter was inserted into the right ureter. Right lower calyceal access was performed with 4.85 Fr all-seeing needle under fluoroscopy while the patient was in the prone position. After visualization of the stone, the three-way adaptor mechanism (irrigation system, microoptic, laser) of micro-PNL was inserted to the proximal end of the needle. Stone fragmentation started by using 272 *μ*m Holmium-Yag laser system. During lithotripsy, infrahepatic accumulation of stone fragments and upper calyceal perforation were detected by fluoroscopy. After detecting this perforation, we have terminated the procedure by leaving the 5F ureteral catheter inside the right ureter for drainage and no nephrostomy tube was placed. On postoperative day 1, patient had right lumbar tenderness and urine leakage from the access tract. No fever was detected during follow-up. We have identified perirenal millimetric stone fragments on kidney, ureter, and bladder X-ray (KUB) and noncontrast computed tomography revealed perirenal urinoma, perirenal and infrahepatic stone fragments, and lower calyceal stone fragments inside the system (Figures 2(a) and 2(b)). According to these findings, we have planned an intervention on postoperative day 2 to drain the perirenal urinoma, in order to avoid further complications. Right lower calyceal access was performed with 20F Amplatz sheath. 12F nephroscope and Holmium-Yag laser were used for stone fragmentation. The operation terminated by placing a double J (DJ) stent in an antegrade fashion and a 14F nephrostomy tube. After the second operation, the right kidney was stone-free and perirenal collection of stone fragments was visible on KUB (Figure 3). After removing the nephrostomy tube, patient was discharged from the hospital on postoperative day 6 of second operation. The DJ stent was removed on 3rd postoperative week.

## 3. Discussion

Micro-PNL is a recently introduced procedure for kidney stones, which has a smaller access tract without the need for serial dilatation. The all-seeing needle (4.85 Fr) with a 3-way adaptor for irrigation, laser, and microoptic system is used during this surgery. Smaller access tract has an advantage of less hemoglobin drop after surgery, when compared to larger tracts used in other PNL types.

Even though ureteral catheter is placed before gaining access to the system, absence of a sheath in micro-PNL limits effective drainage of irrigation fluid and thus increases IPP as mentioned in a recently published article by Tepeler et al. They compared the intrarenal pelvic pressures during conventional and micro-PNL cases and reported that IPP is significantly higher during micro-PNL cases [4]. What we have observed in our case is a very dramatic outcome of this pressure increase. There are certain cases in recently published articles that are examples of this condition. Hatipoglu et al. reported 3 cases [5] and Silay et al. reported 1 case [6], which developed abdominal distention due to extravasation of irrigation fluid. There are certain recommended ways to avoid this complication. Penbegul et al. introduced a new access method using a 14-gauge (6.6 Fr) angiocath for access, in order to leave an available space for drainage between sheath and microoptic system [7]. However Desai made an editorial comment on this article and cited some of his concerns about using a 14-gauge angiocath during micro-PNL. Desai mentioned that angiocaths are collapsible and may not remain patent and drain the fluid efficiently during surgery [8]. Bigger sized ureteral catheter can be used. 7-8 Fr ureteral catheters drain more effectively than other lower sized catheters; thereby, the risk of increase in IPP levels is lower in bigger sized catheters [9]. In our opinion, extra access to pelvicalyceal system combined with ureteral catheterization can be used to decrease the IPP.

There are also some predisposing factors for increased IPP like obstructed ureter (e.g., due to a ureteral stone) and impacted pelvic stone obstructing the drainage. Micro-PNL should be avoided in these situations.

In conclusion, micro-PNL is a safe and efficient method in appropriate sized stones. We wanted to focus on the risk of IPP increase during this surgery. Ineffective drainage due to the absence of a sheath and ineffective drainage capacity of ureteral catheters can be listed as reasons of this undesired effect. More effective drainage methods, without being more invasive, can make micro-PNL method safer and more preferable.
